# Primary gastrinoma of the gallbladder: a case report and review of the literature

**DOI:** 10.3389/fonc.2023.1279766

**Published:** 2024-01-31

**Authors:** Yao-Ge Liu, Shi-Tao Jiang, Yang Zhou, Jun-Wei Zhang, Xin-Ting Sang, Lei Zhang, Xin Lu, Yi-Yao Xu

**Affiliations:** ^1^ Department of Liver Surgery, Peking Union Medical College Hospital, Chinese Academy of Medical Sciences and Peking Union Medical College (CAMS & PUMC), Beijing, China; ^2^ Department of Pathology, Peking Union Medical College Hospital, Chinese Academy of Medical Science and Peking Union Medical College (CAMS & PUMC), Beijing, China

**Keywords:** gastrinoma, gallbladder, Zollinger-Ellison syndrome, neuroendocrine neoplasm, case report

## Abstract

**Background:**

Primary gallbladder gastrinoma is an exceptionally uncommon tumor and is a rare form of neuroendocrine neoplasm. Until now, no cases of primary gallbladder gastrinoma and rare cases of primary gastrinoma from the biliary system have been reported.

**Case presentation:**

We report a case of a 50-year-old woman with watery diarrhea who intermittently received proton pump inhibitors (PPIs) as treatment. A serum gastrin level of 711 pg/ml was recorded after the withdrawal of PPI over 1 week. Enhanced computed tomography (CT) imaging and octreotide imaging uncovered a solitary tumor at the hepatic hilar region. During the laparoscopic surgery, it was determined that the tumor had its origin in the wall of the gallbladder neck, prompting the implementation of a laparoscopic cholecystectomy. Histological analysis revealed a primary neuroendocrine tumor from the neck of the gallbladder. The patient’s symptoms disappeared after the surgery with a follow-up of 6 months.

**Conclusions:**

This case confirmed that primary gallbladder gastrinoma represents a distinct nosological entity. Immunohistochemical analysis plays a pivotal role in the diagnostic process. Given the limited understanding of primary gallbladder gastrinoma, our objective is to offer novel insights into this rare disease by delivering distinctive information and highlighting the therapeutic significance of surgical intervention.

## Introduction

Gastrinoma is the cause of Zollinger–Ellison syndrome (ZES), which is characterized by excessive stomach acid secretion, leading to severe acid-related digestive disorders and diarrhea (1). It has been reported that 80% of gastrinomas were sporadic, while 20%–30% of gastrinomas were associated with multiple endocrine neoplasia type 1 (MEN1) (2, 3). A significant majority (50%–88%) of sporadic ZES cases involve gastrinomas situated within the duodenum (3). The majority of primary gastrinomas are typically found within the conventional gastrinoma triangle, delineated by the junction of the cystic duct and common bile duct, the junction of the second and third portions of the duodenum, and the junction of the neck and body of the pancreas (4). However, rare cases have documented the presence of primary gastrinomas in extrapancreatic and extraintestinal locations within the abdomen, including the lymph nodes, lungs, ovaries, and hepatobiliary tract (5–8). Primary gastrinomas located in the biliary tract are extremely uncommon, and to date, there have been no literature reports on primary gastrinomas originating in the gallbladder. In this report, we present an unusual case of primary gallbladder gastrinoma, reporting the diagnostic process, surgical interventions, and pathological findings.

## Case report

### Clinical presentation

A 50-year-old female patient began experiencing unexplained episodes of watery diarrhea 2 years ago, occurring approximately seven to eight times a day, without complaints of abdominal pain or distension and with no significant body weight loss. She described her stools as having no bloody or mucus elements. Her symptoms showed intermittent improvement with the occasional use of rabeprazole, but there was no systematic treatment in place. In November 2022, she visited our gastroenterology outpatient clinic to undergo gastroduodenoscopy and colonoscopy, which suggested gastric and duodenal ulcers and a negative *Helicobacter pylori* test. After the withdrawal of proton pump inhibitor (PPI) over 1 week, a gastrin level of 711 pg/ml was recorded. This situation has led outpatient physicians to be more inclined to diagnose patients with ZES.

**Figure 1:**
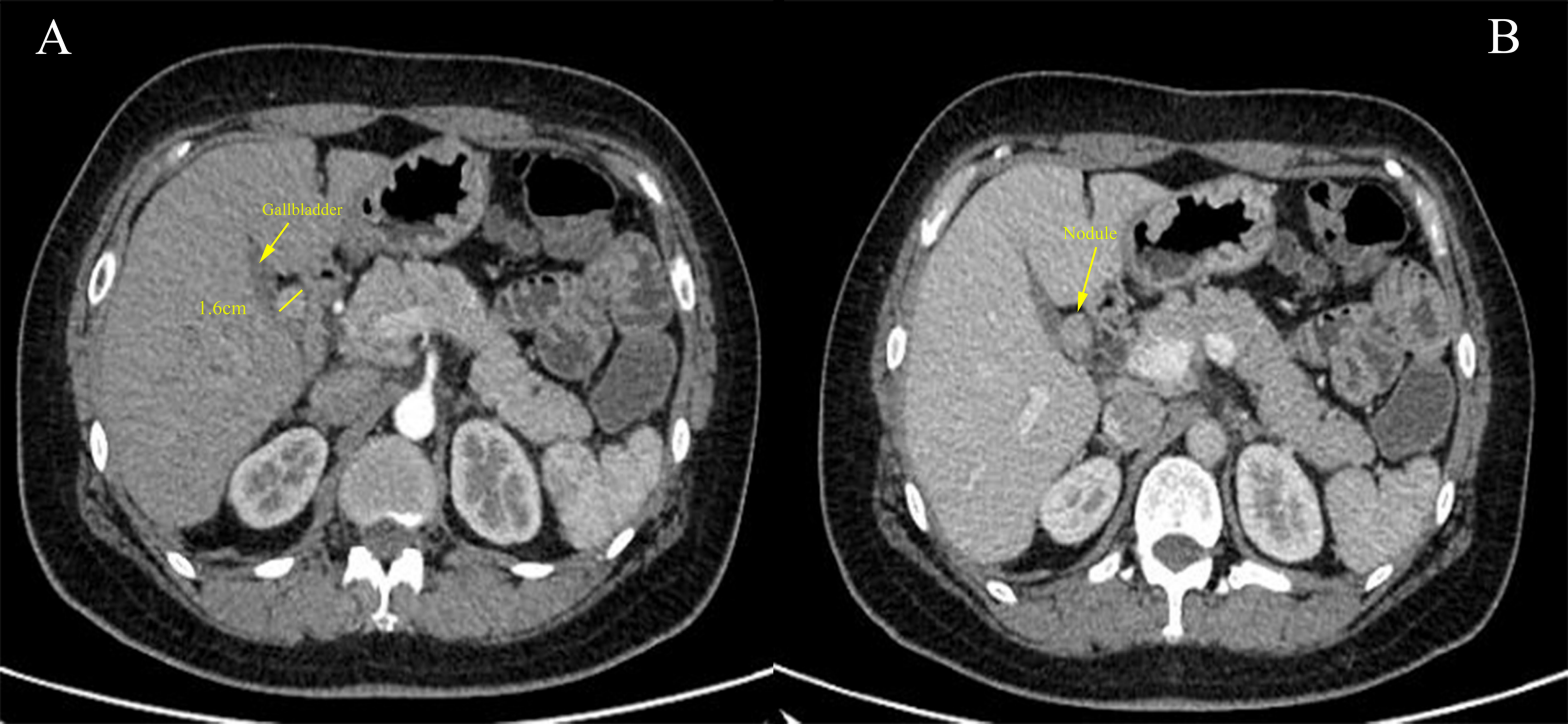
**(A)** Cross-sectional image of the contrast-enhanced abdominal computed tomography (CT) scan demonstrated a 1.6-cm nodule (yellow solid line) adjacent to the gallbladder with significant enhancement during the arterial phase. **(B)** The nodule showed continuous enhancement in the portal phase.

The patient further completed an enhanced abdominopelvic computed tomography (CT) scan, which reported a round-like soft tissue density shadow with a diameter of approximately 1.6 cm in the hepatic hilar region, and the enhanced scan showed significant enhancement in the arterial phase and continuous enhancement to the portal phase (**Figure 1**). Obstruction of the cystic duct and biliary was not found. Octreotide imaging reported high expression of somatostatin receptors in the hepatic hilar region (**Supplementary Figure 1A**). Single-photon emission computed tomography (SPECT)/CT fusion imaging further clarified the localization of the tumor (**Supplementary Figure 1B**). Abdominal ultrasound reported a homogeneous hypoechoic nodule in the hilar region with regular morphology and clear borders. Tumor markers including carcinoembryonic antigen (CEA), carbohydrate antigen 19-9 (CA19-9), alpha-fetoprotein (AFP), neuron-specific enolase (NSE), and pro-gastrin-releasing peptide (proGRP) were screened with negative results. Her serum calcium and parathyroid hormone (PTH) levels were within the normal range. Her liver enzyme levels were normal with an alanine aminotransferase (ALT) level of 24 U/L and an aspartate aminotransferase (AST) level of 25 U/L. Her total bilirubin level was 6.7 μmol/L, with a direct bilirubin level of 2.1 μmol/L. Albumin measured 39 g/L, coagulation function was within the normal range, and there were no indications of hepatic encephalopathy or ascites. Above all, the patient was classified as Child-Pugh class A for liver function. There was no reported family history similar to her condition. Considering the patient’s clinical data, these findings significantly reduce the likelihood of a diagnosis of MEN1. In the patient’s previous medical history, bilateral salpingectomy was performed in March 2022 as a result of a tubo-ovarian abscess. Additionally, the patient has had well-managed hypertension for a duration of 5 years, controlled by a daily administration of 150 mg of irbesartan. The patient denied a family history of gastrointestinal tumors. Above all, gastrinoma was first considered possible. The patient provided consent for an exploratory laparoscopic operation with the potential of hepatic hilar tumor resection and biliary reconstruction.

After preoperative preparation and evaluation, the patient underwent surgery on January 31, 2023. The operation was initially considered exploratory in nature prior to the surgery. We were surprised to find that the nodule was growing entirely from the wall of the gallbladder neck. The tumor was found to be non-invasive, without involvement of the surrounding liver parenchyma or bile duct structures, approximately 2 cm in diameter, and characterized by a smooth and regular surface. No lesion was found in the examined stomach and duodenum. Despite that octreotide imaging could identify small tumor sites (9), it failed to report other sites of high expression in this patient; although we suspected gastrinoma before surgery, no lymphadenectomy or lymph node biopsy was performed during the surgery. Based on intraoperative findings, we ultimately determined to perform a laparoscopic cholecystectomy, during which the gallbladder and the whole tumor were completely excised.

At the time of postoperative day no. 1, the patient’s serum gastrin level was normal at 17 pg/ml. The patient had a good postoperative recovery and did not experience any post-surgery complications. Due to the patient’s preoperative clinical symptoms, a more conservative transitional dietary strategy was implemented postoperatively, and the patient was discharged on the fourth postoperative day. The patient accepted follow-up from the outpatient service in our hospital, with the monitoring of serum gastrin levels and symptoms. On April 23, 2023, she tested serum gastrin with a level of <10 pg/ml. After a 6-month postoperative follow-up, the patient did not show a recurrence of gastrointestinal symptoms and was able to discontinue PPIs after surgery. In the future, we will maintain long-term follow-up and advise the patient to accept an abdominal ultrasound examination per year.

### Pathological findings

**Figure 2:**
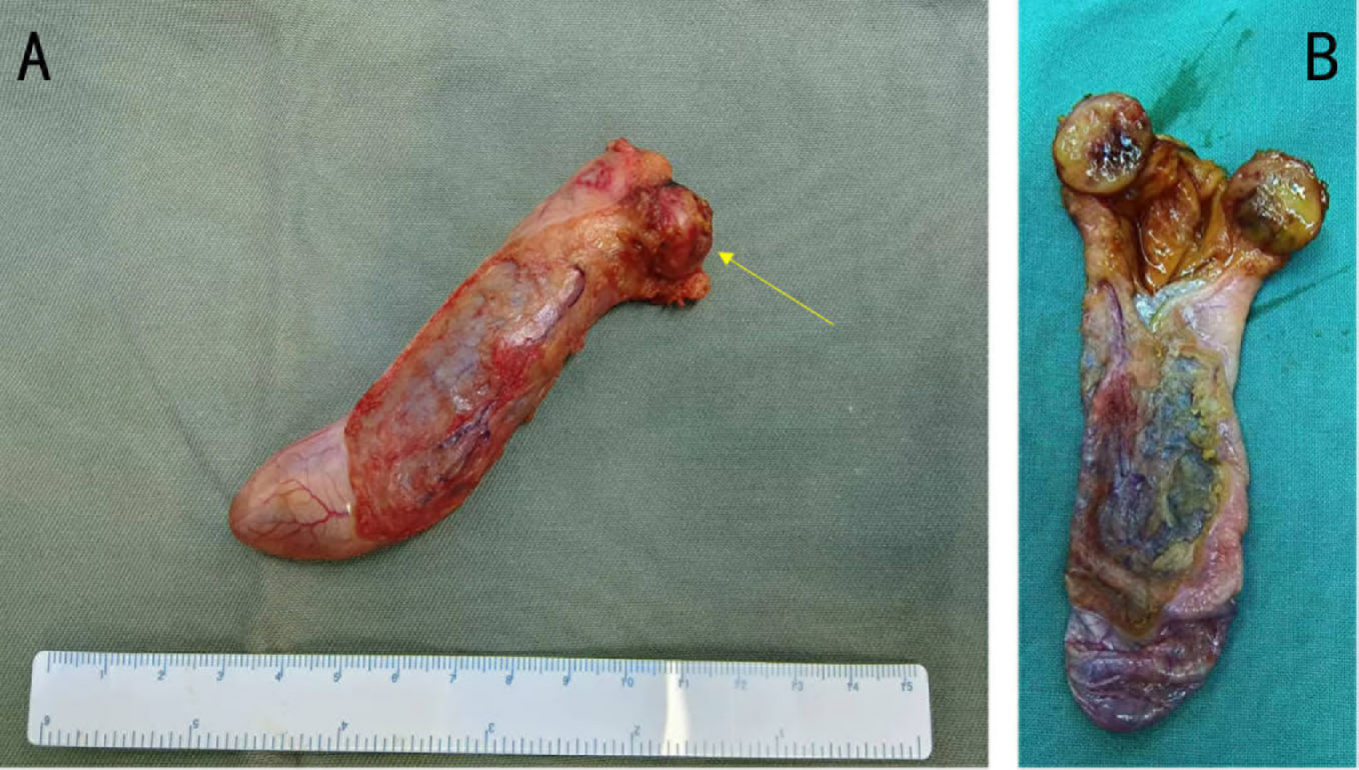
**(A)** Gross photograph of the tumor (yellow arrow) and gallbladder. **(B)** The tumor together with a portion of the gallbladder has been sectioned, revealing a close relationship between the tumor and the gallbladder. The tumor is located in the neck of the gallbladder and near the opening of the cystic duct.

**Figure 3:**
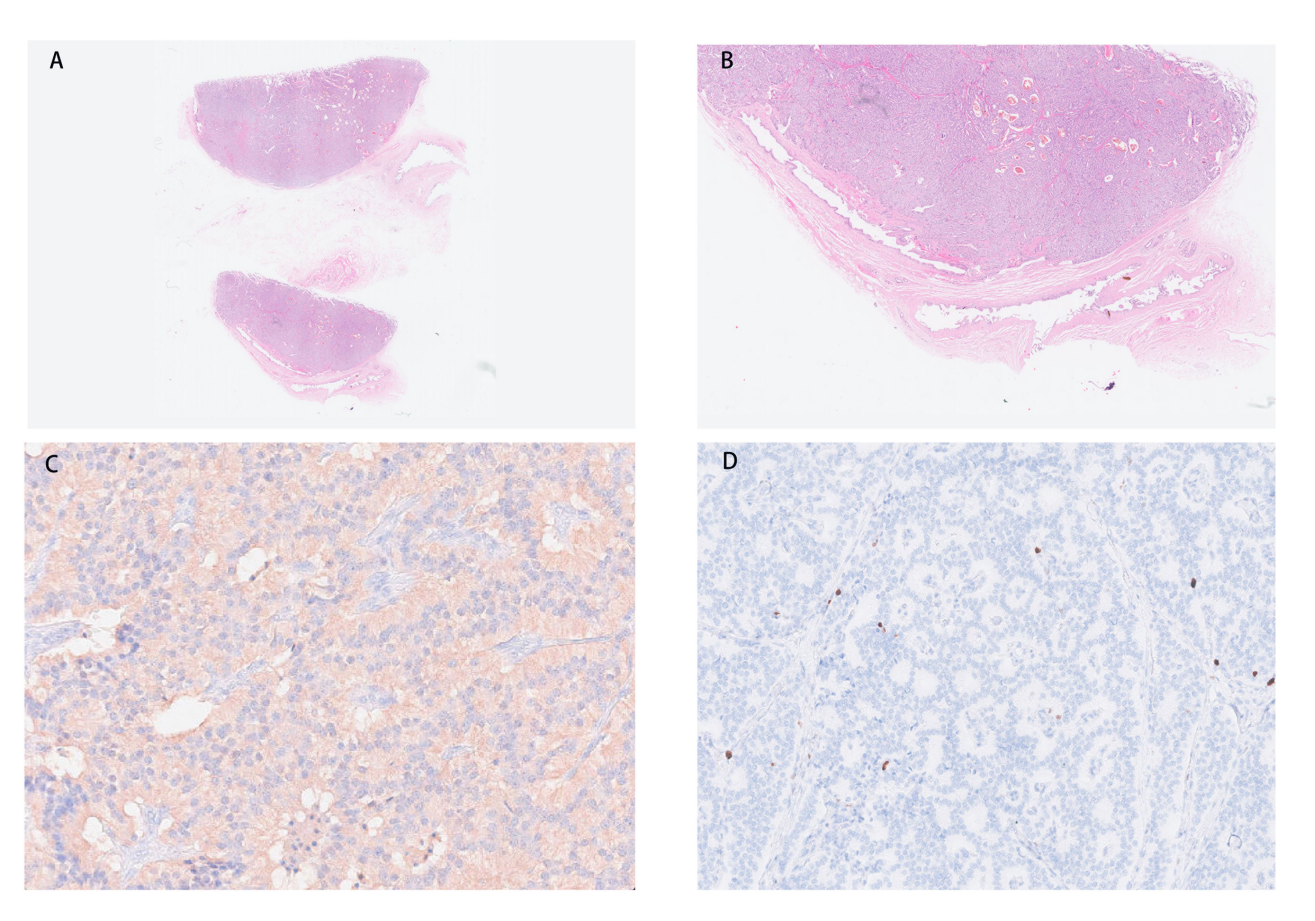
**(A, B)** The tumor is located in the muscular layer of the gallbladder. **(C)** Immunohistochemical staining for gastrin shows strong and diffuse positive in tumor cells (magnification, ×200). **(D)** NET with a low Ki-67 proliferation index (magnification, ×100). NET, neuroendocrine tumor.

In the macroscopic view of the tumor shown in **Figures 2A, B**, a smooth tumor was located in the neck of the gallbladder. The longitudinal section of the mass can help to better observe the relationship between the tumor and gallbladder. The examination of immunohistochemistry performed on paraffin-embedded sections confirmed a primary neuroendocrine tumor originating from the gallbladder, with 2% of cells staining positive for Ki-67 (**Figure 3D**) and mitotic figures <2 for 10 HPF. Microscopic examination confirmed the source of the tumor, which was located in the muscular layer of the gallbladder (**Figures 3A, B**). Immunohistochemical staining for gastrin showed strong positive and diffuse positive staining in tumor cells (**Figure 3C**). In light of the pathological findings, the diagnosis of a primary neuroendocrine tumor (WHO grade 1) was made.

## Discussion

Gastrinoma is the second most common functional neuroendocrine tumor (NET) that leads to ZES (1). The incidence of gastrinoma is 0.5–3/million population per year (10). Most previously reported gastrinomas of hepatobiliary tract origin were from the hepatic parenchyma (11) or the bile duct. However, to our knowledge, we have reported the first case of primary gallbladder gastrinoma. Until now, only six pieces of literature describing 10 cases of gastrinomas that originate from the biliary tract have been reported in the English literature (8, 12–16), including two cases with MEN1 reported by Price et al. (15).

Gastrinomas of the biliary tract can originate from different parts of the biliary system (**Table 1**). The majority of reported cases were female. The tumors were usually small in size, as six reported cases have diameters ranging from 0.6 cm to 4.0 cm. Apart from showing common gastrointestinal symptoms due to hypersecretion of gastric acid, patients may exhibit symptoms of jaundice due to tumor compression (12). All the reported cases underwent surgical resection and were followed up for a period ranging from 4 months to 7 years. Most of the cases showed no evidence of recurrence and demonstrated a favorable surgical outcome. Only one patient died due to gastrinoma, as lymph node metastasis was confirmed by surgery, and the disease recurred 4 years after the surgery. She then received long-acting release octreotide treatment, radiation therapy targeting bone metastatic lesions, and ^111^In-labeled octreotide infusion therapy and passed away in the seventh year after the surgery.

**Table 1:** Reported cases of biliary tract gastrinomas including symptoms, sites, sizes, gastrin levels, follow-up periods, and outcomes.

Reference, year	Cases	Sex/age (years)	Symptoms	Site	Size (cm)	Gastrin level before surgery	Follow-up period (months)	Outcome
Mandujano-Vera et al., 1995 (12)	1	F/53	Nausea, pyrosis, AP	CBD	2.2	90 ng/ml	8	No recurrence
Martignoni et al., 1999 (13)	1	M/60	Diarrhea, vomiting, GRD	CHD	1.3	1,768 pg/ml	36	No recurrence
Tarcin et al., 2011 (14)	1	F/44	Nausea, diarrhea	CBD	8	>65,000 pg/ml	4	No recurrence
Norton et al., 2018 (8)	3	NA	NA	1 CHD 1 LHD 1 RHD	NA	NA	NA	NA
Price et al., 2009 (15)	1	F/55	NA	Junction of CD and CBD	0.6	>4,500 pg/ml	24	No symptom
Price et al., 2009 (15)	1	F/43	GRD	CBD	1.5	360 pg/ml	24	No recurrence
Price et al., 2009 (15)	1	F/28	Diarrhea, AP, GRD	LHD	4.0	1,600 pg/ml	84	Died due to the disease
Wu et al., 1997 (16)	1	M/54	NA	CBD	1.5	1,867 pg/ml	5	No recurrence
Present case	1	F/50	Diarrhea	Gallbladder	1.6	711 pg/ml	6	No recurrence

NA, not available; GRD, gastroesophageal reflux disease; AP, abdominal pain; CBD, common bile duct; CHD, common hepatic duct; LHD, left hepatic duct; RHD, right hepatic duct; CD, cystic duct.

Although case reports on sporadic primary gastrinoma originating from the biliary system are extremely rare, large-scale clinical cohorts have provided references for the incidence rate of the disease (8, 16). Norton et al. retrospectively reported four primary liver and three primary biliary tract tumors in 223 patients who underwent surgery to remove gastrinomas without MEN1 and described the prognosis of rare gastrinomas originating from the hepatobiliary tract (8). The result showed that four patients (57%) with long-term follow-up had recurrent disease, and three patients (43%) were identified with portal lymph node metastases during the surgery. They recommended that lymph nodes in the porta hepatis should be routinely excised. However, the rarity of the disease limited the spread of surgical approach recommendations. A similar study conducted by Wu and colleagues also reported one primary biliary tract gastrinoma and three primary liver gastrinomas among 215 patients who were definitively diagnosed with ZES (16). Regarding the approximate incidence of primary biliary tract gastrinomas as 1% in these two cohorts, we hypothesized that there might be more primary biliary gastrinomas that have not been well documented.

The diagnosis of resected primary biliary tract gastrinoma may be difficult to explain (17). First of all, primary biliary tract gastrinoma could be confused with lymph node metastases or even primary lymph node gastrinoma. In terms of location, the biliary duct goes through the gastrinoma triangle, which contains abundant lymph nodes, and approximately 85% to 90% of gastrinomas are located (18). From an embryological perspective, one theory explains that in the lymph nodes around the pancreas, there may occasionally be differentiated neuroendocrine cells capable of producing gastrin-secreting cells (19). This provides theoretical support for primary lymph node gastrinomas. Similarly, existing hypotheses to explain primary biliary gastrinomas include multipotential endocrine cells in the biliary system, ectopic pancreatic tissue in the bile duct, and metaplastic epithelium in the biliary tree (12). Therefore, it is worth further investigating whether independently originated neuroendocrine cells exist within the biliary tract system, which could potentially provide a theoretical basis for primary biliary gastrinomas. However, despite that the use of highly sensitive methods such as somatostatin receptor imaging and endoscopic ultrasound ultrasonography can aid in the precise identification of tumors, they often fail to detect most small duodenal tumors (1–5 mm) in 60%–80% of patients with ZES (8, 9, 17, 20, 21). This suggested that small duodenal tumors may be primary rather than other ectopic tumors with greater size. However, in one clinical trial conducted by Cadiot and colleagues, traditional techniques such as CT scans and endoscopic ultrasonography individually exhibited sensitivities of 58% for tumor detection, while Octreoscan scintigraphy yielded a similar sensitivity of 58%. However, when used in combination, their collective sensitivity reached 90% in accurately identifying tumors (9). They also found that Octreoscan scintigraphy could identify duodenal gastrinoma as small as 3 mm (9). This suggests that the combined use of multiple imaging studies can reduce the misdiagnosis rate of small gastrinomas.

Sporadic gastrinoma with no evidence of metastasis should undergo exploratory laparotomy and radical surgical resection (8). In our report, as the multiple imaging results of the patient excluded the possibility of a primary tumor in other sites or any distant lymph node metastasis, and in conjunction with the patient’s excellent postoperative recovery during a six-month follow-up, we have compelling grounds to assert that the gastrinoma originated in the gallbladder and that the surgery was curative.

As demonstrated by the presented case, we possess strong justification to support the notion that primary gallbladder gastrinoma represents a distinct nosological entity that deserves further attention. The histogenesis of endocrine tumors in the gallbladder remains unclear. Further research is needed to explore the tumor origin of primary gallbladder gastrinoma from a genetic perspective.

## Data availability statement

The original contributions presented in the study are included in the article/**Supplementary Material**. Further inquiries can be directed to the corresponding authors.

## Ethics statement

The studies involving humans were approved by Peking Union Medical College Hospital. The studies were conducted in accordance with the local legislation and institutional requirements. The participants provided their written informed consent to participate in this study. Written informed consent was obtained from the individual(s) for the publication of any potentially identifiable images or data included in this article.

## Author contributions

Y-GL: Conceptualization, Resources, Writing – original draft, Writing – review & editing. S-TJ: Writing – original draft. YZ: Writing – original draft. J-WZ: Writing – review & editing. X-TS: Writing – review & editing. LZ: Writing – review & editing. XL: Conceptualization, Writing – review & editing. Y-YX: Conceptualization, Formal analysis, Writing – review & editing.
