# Supplementary material for: Primary gastrinoma of the gallbladder: a case report and review of the literature
Source: Front Oncol. 2024 Jan 31;13:1279766. doi: 10.3389/fonc.2023.1279766 (PMC10864482; doi:10.3389/fonc.2023.1279766)
Supplement: Supplementary file 1 [file DataSheet_1.docx]

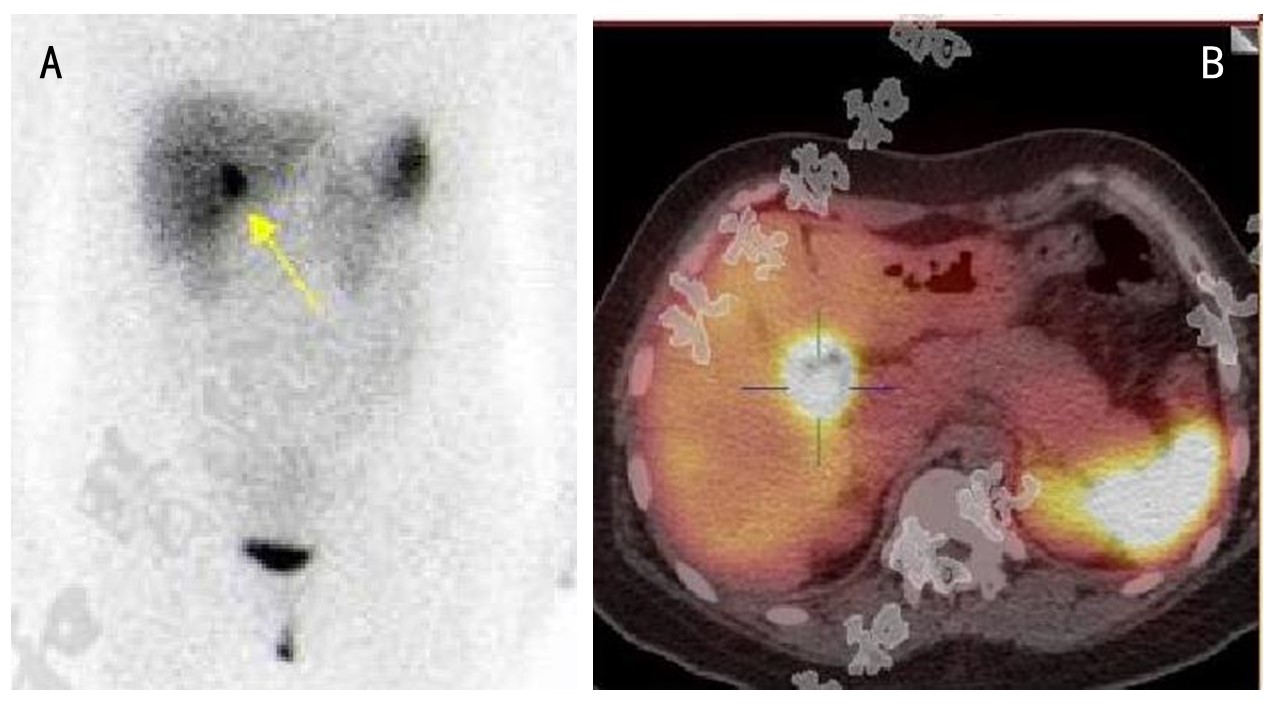


**Supplementary Figure 1 a** Octreotide imaging showed increased uptake at the hepatic hilum (yellow arrow) with no significant uptake observed in other abdominal regions. **b** SPECT-CT imaging indicated the location of the high uptake mass corresponding to the findings on the contrast-enhanced CT
